# Sonication of heart valves detects more bacteria in infective endocarditis

**DOI:** 10.1038/s41598-018-31029-w

**Published:** 2018-08-28

**Authors:** Anna Gomes, Marleen van Oosten, Kasper L. B. Bijker, Kathleen E. Boiten, Elisa N. Salomon, Sigrid Rosema, John W. A. Rossen, Ehsan Natour, Yvonne L. Douglas, Greetje A. Kampinga, Sander van Assen, Bhanu Sinha

**Affiliations:** 1 Department of Medical Microbiology and Infection Prevention, University of Groningen, University Medical Center Groningen, Groningen, The Netherlands; 2 Department of Thoracic Surgery, Maastricht University Medical Center, Maastricht, The Netherlands; 3 Department of Cardio-Thoracic Surgery, University of Groningen, University Medical Center Groningen, Groningen, The Netherlands; 4 Department of Internal Medicine (Infectious Diseases), Treant Zorggroep, Hoogeveen, The Netherlands

## Abstract

Optimal antimicrobial treatment of infective endocarditis requires identification and susceptibility patterns of pathogens. Sonication of explanted heart valves could increase the identification and culture of pathogens, as shown in prosthetic joint and pacemaker/ICD infections. We tested 26 explanted heart valves from 20 patients with active definite endocarditis for added diagnostic value of sonication to the standard microbiological workup in a prospective diagnostic proof of concept study. Two sonication protocols (broth enrichment *vs*. centrifugation) were compared in an additional 35 negative control valves for contamination rates. We selected sonication/centrifugation based on acceptable false positive rates (11.4%; 4/35). Sonication/enrichment yielded many false positive results in negative controls (28.6%; 10/35), mainly *Propionibacterium acnes* (next-generation sequencing excluded technical problems). Compared to direct culture only, adding sonication/centrifugation (including molecular testing) significantly increased the diagnostic yield from 6/26 to 17/26 valves (p = 0.003). Most importantly, culture positives almost doubled (from 6 to 10), providing unique quantitative information about antimicrobial susceptibility. Even if direct molecular testing was added to the standard workup, sonication/centrifugation provided additional diagnostic information in a significant number of valves (8/26; 31%; p = 0.013). We concluded that sonication/centrifugation added relevant diagnostic information in the workup of heart valves with infective endocarditis, with acceptable contamination rates.

## Introduction

Early and accurate diagnosis is crucial in infective endocarditis, as delay in treatment negatively affects clinical outcome and costs^1–3^. Determination of the causative microorganism and its resistance pattern is a prerequisite for implemention of appropriate antimicrobial therapy^4^. However, microbiological results in the detection of intracardiac infection are false-negative in 10% to 49% of the cases^5–7^. False-negatives result from biofilm formation in endocarditis and prior antimicrobial therapy, similarly to prosthetic joint infections^8–10^. To mobilize bacteria from biofilm, sonication is used as a method through application of ultrasonic waves^4–6,11,12^. Importantly, bacterial cell structures are not significantly damaged and remain viable for culture under appropriate conditions^13^.

Culture of sonication fluid was shown to be more sensitive than standard culture of tissue surrounding orthopedic prostheses, especially in patients who received antimicrobial treatment within 14 days before surgery^12,14–18^. Therefore, sonication is now regarded as essential add-on to standard microbiological workup in prosthetic joint infections^5,6,11,12,14–17,19^.

The clinical usefulness of sonication has also been shown for the microbiological diagnosis of pacemaker and implantable cardioverter defibrillator related infections^4–6,11,20–22^. Culture after sonication of explanted pacemakers and implantable cardioverter defibrillators was more sensitive than traditional culture (54–94% versus 27–56%), and presurgical antimicrobial therapy did not reduce its diagnostic performance^4–6,21,22^. Sonication was shown to be the only method detecting pathogens in 24% of cases, and traditional cultures confirmed pathogens detected by sonication when both methods were positive^4^.

We hypothesized that sonication of heart valves will increase the identification and culture of pathogenic microorganisms in infective endocarditis, as compared to the standard microbiological workup. Furthermore, we hypothesized that sonication may also lead to over-isolation of contaminating bacteria. Therefore, valves of patients with definite active endocarditis according to expert judgement were analysed to investigate the value of two sonicaton protocols in the identification of pathogenic microorganisms. Additionaly, negative control valves were analysed to investigate the methodological contamination error of these two sonication protocols. Endpoints were identification and culture of microorganisms by sonication, as addition to standard microbiological workup with or without molecular testing using 16S ribosomal DNA PCR (16S-PCR).

## Methods

### Study design, patient population, and specimen collection

**Figure 1:**
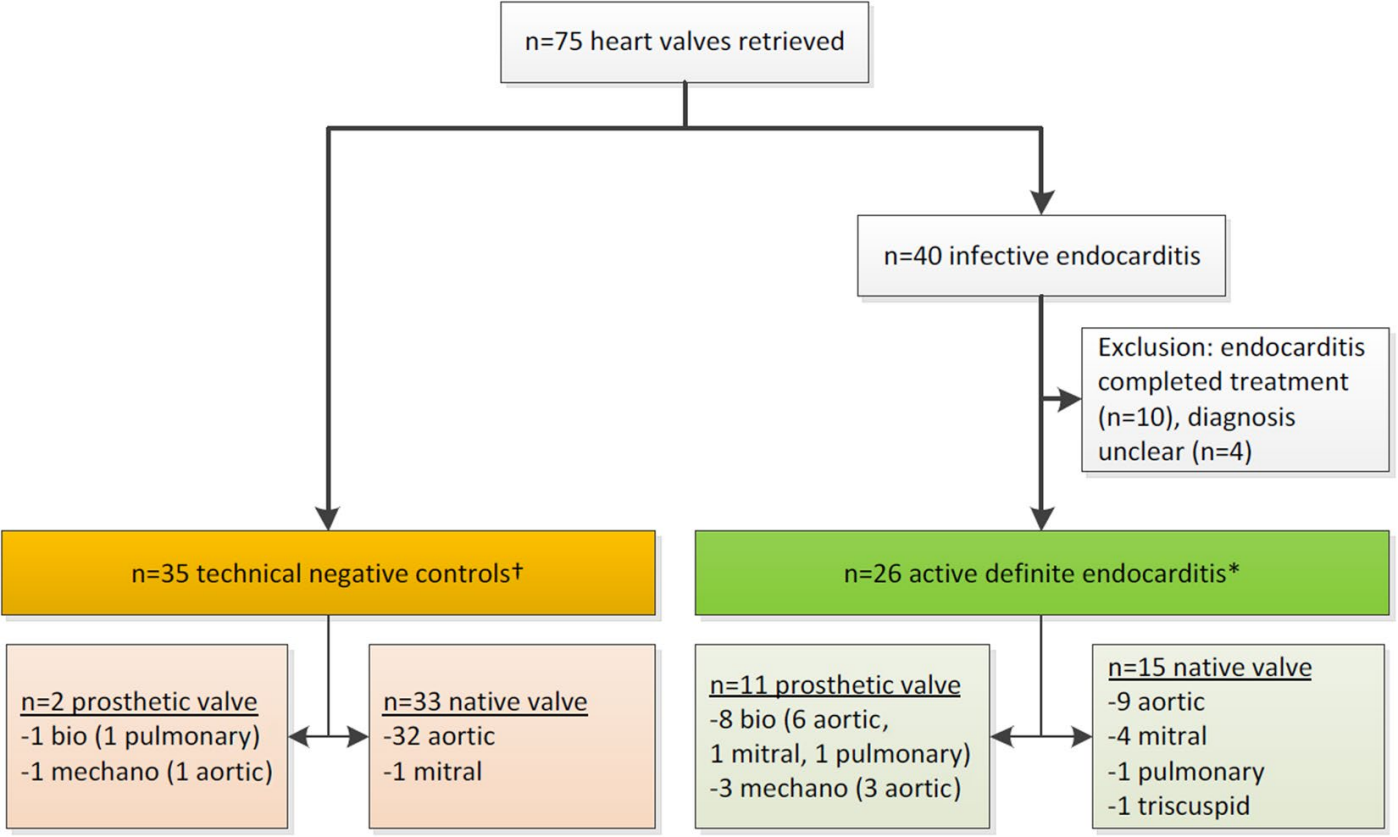
Inclusion and exclusion of heart valves in study. Bio = biological prosthetic valve; mechano = mechanical prosthetic valve; n = number. *Diagnosis of active definite endocarditis was made for n = 23 valves presurgically (on antimicrobial therapy for a median duration of 27 [range 4–54] days) and for n = 3 valves surgically (antimicrobial therapy started thereafter); ^†^Surgical indication of negative controls: valves stenosis (n = 33), valve insufficiency (n = 1), valve prosthesis too small due to growth of patient (n = 1).

**Figure 2:**
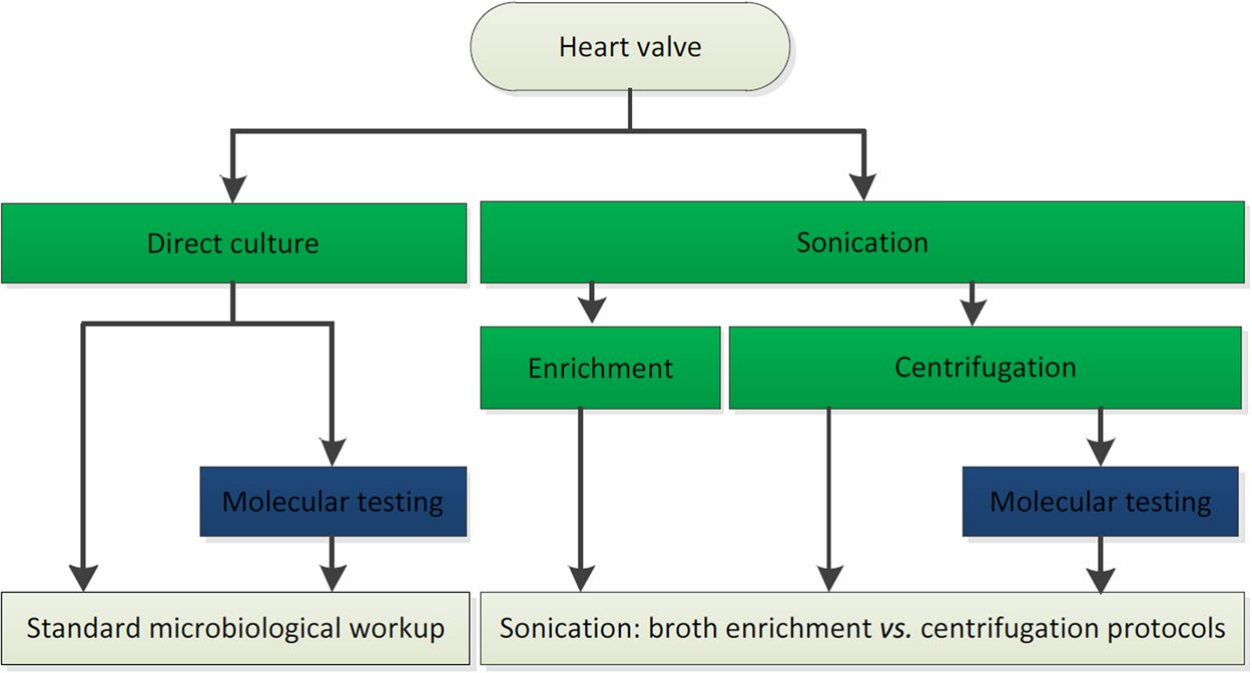
Microbiological workflow of heart valves (active endocarditis and negative controls). Centrifugation = centrifugation of sonication fluid and thereafter culture of the sediment on solid media - BA, CHOC, and BBA agar plates; Direct culture = pressing of aberrant looking parts of the valve onto the solid media BA, CHOC, and BBA plates; Enrichment = direct culture of sonication fluid on solid media - BA, CHOC, and BBA agar plates - as well as enrichment of sonication fluid in blood culture bottles; Molecular testing (direct) = in this study comprising 16S-PCR performed directly on aberrant looking parts of the valve; Molecular testing (sonication) = in this study comprising 16S-PCR performed on the sediment of sonication fluid retrieved after centrifugation; Sonication = the sonication procedure of the explanted heart valve. Hereafter, the sonication fluid was handled according to two different protocols: enrichment and centrifugation; Standard = standard workup, including Gram-stain and direct culture on the solid media BA, CHOC, and BBA agar plates.

A diagnostic proof of concept study with prospective inclusion was performed, investigating 61 heart valves from 55 subjects. These subjects underwent heart valve replacement for non-infection related hemodynamic failure (35 valves from 35 subjects) or presumed infective endocarditis (26 valves from 20 subjects) at the University Medical Center Groningen, the Netherlands, and were enrolled between April 15, 2016, and March 1, 2017 (Fig. 1). Both native and prosthetic valves were included. Standard microbiological workup (with or without molecular testing) and two different sonication protocols (broth enrichment *vs*. centrifugation) of heart valves were compared (Fig. 2). The medical ethical review board of the University Medical Center Groningen approved the study (review board reference number M16.186555 and subject METc 2015/552). The research was performed in accordance with the relevant guidelines and regulation of this review board. Written informed consent was obtained from each patient.

Heart valves were separately collected in sterile jars and dryly transported to the microbiology laboratory for direct processing. Heart valves of patients undergoing valve replacement for hemodynamic failure of non-infectious origin were randomly selected and included as negative controls. Heart valves of patients undergoing valve replacement for a “definite” active infective endocarditis according the modified Duke criteria^23^ and expert clinical judgement were consecutively included to investigate the added clinical value of sonication to the standard microbiological workup. Active disease was defined as the episode for which patients were on, or about to start, therapeutic antimicrobial treatment. Prior antimicrobial therapy was defined as receipt of antimicrobials within 14 days prior to surgery.

For every diagnostic method in this study, any identification of microoganisms in Gram-stain, 16S-PCR, or culture (≥1 colony forming unit (CFU) or ≥1 blood culture bottle) was regarded positive. The referent standard for microbiological diagnosis included presurgical blood cultures, prefereably before the start of antimicrobial therapy, and standard microbiological workup of the valve. In case of unclear results, diagnostic steps were repeated for confirmation. Growth in cultures of negative controls was regarded as false-positive (“contaminants”).

### Standard (microbiological) workup

In a flow cabinet using sterile gloves, aberrant looking parts of the valve (including vegetations, abscesses, (pseudo)aneurysms, etc.) were identified, excised, and preferably used for direct culture. These pathologic parts were pressed onto blood agar (containing 5% sheep blood; BA), chocolate agar (CHOC), and *Brucella* blood agar (containing 5% sheep blood; BBA) plates (all obtained from Mediaproducts BV, Groningen, Netherlands). Next, valves as a whole were rolled on these agar plates.

### 16S rDNA sequencing

The pathologic parts selected for culture in the standard workup were consecutively used for 16S-PCR (Fig. 2). DNA was extracted using the DNeasy Blood&Tissue-kit according to the manufacturer’s protocol (Qiagen, Hilden, Germany). DNA was subsequently amplified by PCR and sequenced using the forward and reverse primers 8F (5′-TGGAGAGTTTGATCCTGGCTCAG-3′) and 515R (5′-TACCGCGGCTGCTGGCAC-3′), respectively (Biolegio, Nijmegen, Netherlands). PCR was performed for 15 minutes at 95 °C, with 35 cycles of 15 seconds at 94 °C, 15 seconds at 60 °C, 30 seconds at 72 °C, and a final extension at 72 °C for 10 minutes. Sequencing was performed with an automated DNA sequencer (ABI 3500XL; Applied Biosystems Instrument, Carlsbad, CA, USA) using the BigDye Terminator v3.1 cycle sequencing kit. The sequence data was analysed by assembling the forward and reverse sequences into a consensus sequence using SeqMan Pro v10.0.1 (DNASTAR, Madison, WI, USA). The consensus sequence was compared with GenBank sequences using the basic local aligment search tool (BLAST)^24^.

### Sonication (enrichment *vs.* centrifugation)

Heart valves underwent sonication after direct culture and molecular testing was performed (Fig. 2). Specimens were placed in separate sterile (Gamma on 8 k-gray) double packed 0.36-litercontainers (Incense article number 550676, Beldico, Duiven, Netherlands). Sterile Ringer’s solution was added until the sample was completely covered (minimally 140 ml). The container was then vortexed for 30 seconds (IKA® Vortex Genius 3), subjected to sonication for 1 minute (40 kHz, power density 100%, HF power max. 200 W; BactoSonic® ultrasonic bath BS14.2, BANDELIN Electronic GmbH&Co.KG), and vortexed for an additional 30 seconds. The outside of the container was decontaminated with 70% ethanol. Two aliquots of the sonication fluid were further processed (Fig. 2).

For sonication with enrichment, 100 µL aliquots of sonication fluid were plated with a Drigalski spatula onto BA, CHOC and BBA agar plates. Additionally, blood culture bottles (BD BACTEC^TM^) were inoculated with 10 ml fluid using a sterile syringe.

For sonication with centrifugation, a total of 50 ml of fluid was centrifuged at 2500 RCF (3500 rpm) for 15 minutes (Hettich Rotina 46 R centrifuge, Gemini BV) and the supernatant was discarded. 100 µL aliquots of resuspended sediment were plated with a Drigalski spatula onto BA, CHOC and BBA agar plates. In addition, 200 µL was used to perform 16S-PCR.

### Incubation of cultures

BA and CHOC agar plates were incubated at 35 °C under aerobic conditions with 5% CO_2_, and BBA agar plates were anaerobically incubated at 35 °C. All agar plates were incubated for up to 9 days. Blood culture bottles were incubated for 9 days in the BACTEC^TM^ (BD Instrumented Blood Culture Systems). All cultured microorganisms were enumerated. Identification was performed by routine microbiological techniques.

### Next-Generation Sequencing (NGS)

To investigate the genetic relationship between strains of *Propionibacterium acnes* (genus *Cutibacterium*), we performed whole genome sequencing (WGS) on cultured isolates by standard workup or sonication.

DNA was extracted using the UltraClean microbial DNA isolation kit according to the manufacturer’s protocol (MO BIO laboratories, Carlsbad, CA, USA). The DNA library was prepared using the Nextera XT kit (Illumina, San Diego, CA, US) and sequenced on the MiSeq (Illumina) for creation of paired-end 300 base pair reads aiming at coverage of at least 60-fold, as described previously^25,26^. *De novo* assembly was performed using CLC Genomics Workbench v10.0.1 (CLC bio A/S, Aarhus, Denmark) after quality trimming (Qs ≥ 28)^26^. Determination of the phylogenetic relationship was performed with a gene-by-gene approach using SeqSphere^+^ v3.5.2 (Ridom, Münster, Germany)^26^. The genome of *P*. *acnes* strain HL096PA1 (accession number NC_021085.1) was used as a reference, with an additional ten strains as query genomes to extract open reading frames from the genome. cgMLST Target definer (SeqSphere^+^ v1.4) was used to develop an *ad hoc* core genome multi-locus sequence typing scheme^26^. A numerical allele type was assigned by SeqSphere according to the sequence identity of each open reading frame. SeqSphere used the allelic profile formed by the combination of all alleles in each genome for constructing the minimum-spanning tree. This Whole Genome Shotgun bioproject has been deposited at DDBJ/EMBL/GenBank under the accession number PRJEB20613.

### Power analysis

The expected difference between the detection and culture of microorganisms by the two microbiologic techniques (standard workup *vs*. standard workup with sonication) to detect the cause of endocarditis was 30%, based on literature of prosthetic joint infections^12^. Testing two-sided with paired samples, with α = 0.05, β = 0.20 and a power of 0.80, showed a minimum of 16 heart valves to confirm our hypothesis.

We hypothesized that sonication may also lead to over-isolation of (contaminating) bacteria. Therefore, we planned to test control valves from patients not suspected of endocarditis. Since we found *P*. *acnes* in many of these control valves, we doubled the inclusion of this group to 35 in order to get a fairly reliable estimate of contamination due to sonication.

### Statistical analysis

Positive results by the microbiological methods (standard workup *vs*. standard workup with sonication) were compared by two-sided McNemar’s testing of paired proportions. A p-value < 0.05 was considered statistically significant.

## Results

### Study population and explanted heart valves

75 valves were retrieved, of which 14 were excluded since diagnosis of definite endocarditis was unclear or antimicrobial treatment was completed (Fig. 1). Finally, 61 valves from 55 patients were included: 35 valves from 35 patients included as negative controls and 26 valves from 20 patients with active definite endocarditis (Table 1).

**Table 1:** Study population.

	Active endocarditis	Controls
Number of patients, n	20	35
Male sex, n (%)	15 (75)	22 (62.9)
Age, years, median [range]	57 [14–80]	71 [17–83]
Number of valves, n	26	35
Native valves, n (%)	15 (58)	33 (94)
- aortic, n (%)	9 (60)	32 (97)
- mitral, n (%)	4 (27)	1 (3)
- pulmonary, n (%)	1 (7)	0
- tricuspid, n (%)	1 (7)	0 (0)
Prosthetic valves, n (%)	11 (42)	2 (6)
Biological, n (%)	8 (73)	1 (50)
- aortic, n (%)	6 (75)	0 (0)
- mitral, n (%)	1 (13)	0 (0)
- pulmonary, n (%)	1 (13)	1 (100)
Mechanical, n (%)	3 (27)	1 (50)
- aortic, n (%)	3 (100)	1 (100)
Median antimicrobial treatment prior to valve removal, days [range]*	27 [4–54]^†^	0

n = number; *all patients received prophylactic antibiotics during surgery (standard: cefazoline 3 × 2 gram for 24 hours i.v., with additional doses intraoperatively in case of blood loss >1L, and after 4 hours operation time); ^†^three patients did not receive antimicrobial therapy before valve removal.

### Investigating the methodological contamination error of sonication in negative control valves

**Figure 3:**
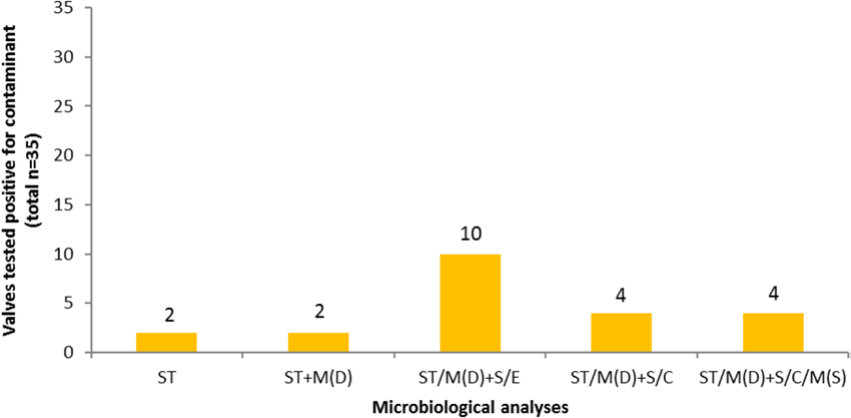
Negative control valves (n = 35 in total) with the identification of contaminating microorganisms by various microbiological analyses. C = centrifugation of sonication fluid and thereafter culture of the sediment on solid media - BA (blood agar +5% sheep blood), CHOC (chocolate agar), and BBA (*Brucella* blood agar +5% sheep blood) agar plates; E = enrichment, including direct culture of sonication fluid on solid media (BA, CHOC, and BBA plates) as well as enrichment of sonication fluid in blood culture bottles; M(D) = molecular testing, in this study comprising 16S-PCR performed directly on aberrant looking parts of the valve; M(S) = molecular testing, in this study compromising 16S-PCR performed on the sediment of sonication fluid retrieved after centrifugation; S = sonication, includes the sonication procedure of the explanted heart valve, hereafter the sonication fluid was handled according to two different protocols (enrichment and centrifugation); ST = standard work-up, including direct culture on solid media (BA, CHOC, and BBA plates).

In negative control valves, 16S-PCR never yielded positive results (Supplementary Table S1). In contrast, in direct culture 2/35 (5.7%) valves became positive. Sonication/centrifugation and sonication/enrichment yielded 4/35 (11.4%) and 10/35 (28.6%) false positive results, respectively (Fig. 3). Notably, positive results for sonication/enrichment were mainly caused by isolated growth in blood culture bottles (7/10, Supplementary Table S1). None of the negative control valves revealed signs of infection during macroscopic pathological examination peroperatively (Supplementary Table S2). All negative control patients had an unremarkable follow-up (median 13.8 and range 8.5–15.9 months) without any sign of endocarditis, except for one unrelated case.

*P*. *acnes* was the most commonly isolated microorganism in negative control valves (total 7/35). However, for sonication/centrifugation this species was less frequently found (2/35). Furthermore, if *P*. *acnes* was found in negative control valves after sonication/centrifugation, it was present in only one culture medium with low growth density (1 CFU). For sonication/enrichment, *P*. *acnes* was likewise found in only one culture medium with low growth density but with two exceptions: in the first more culture media were positive, in the second high growth density was found.

Based on the above presented comparison of the two sonication protocols (broth enrichment *vs*. centrifugation) in negative control valves, we chose to focus the remaining results on the protocol of sonication/centrifugation. Supplementary Table S1 also shows the added diagnostic value of sonication/enrichment to the standard microbiological workup in the first n = 17 valves from active definite endocarditis patients.

### Investigating the value of sonicaton/centrifugation in the identification of pathogenic microorganisms in valves of active endocarditis patients

Sonication/centrifugation added extra information for microbiological diagnosis of infective endocarditis in 8/26 of valves, as compared to standard workup with molecular testing (p = 0.013). In patient 3, the pathogen was identified only by 16S-PCR on sonication fluid (*Haemophilus parainfluenzae* on a native tricuspid valve, Table 2). In patients 4 and 29 and 58, sonication provided the isolates (culture) of molecularly identified pathogens directly on the valve (*P*. *acnes* on one biological and two mechanical prosthetic aortic valves, respectively). In patients 30 and 52, results of sonication provided additional proof of the identified microorganism as pathogen (*P*. *acnes* and *Staphylococcus capitis* on a biological prosthetic aortic valve; *P*. *acnes* as second pathogen on a native aortic valve and as only pathogen on a biological prothetic mitral valve). In patient 46, sonication provided both the first identification and culture of the pathogen (*P*. *acnes* on a biological prosthetic aortic valve). Of these 8 heart valves, 7 came from patients receiving antimicrobial therapy prior to surgery (n = 1 detection by 16S-PCR, n = 6 culture on agar plates) and 1 came from a patient who did not receive prior antimicrobial therapy (n = 1 culture on agar plates). Moreover, with the additional information provided by sonication/centrifugation, the duration of therapy was determined in one out of 20 patients. Detailed information about the outcome of culture and 16S-PCR in positively tested heart valves from patients with active definite endocarditis (26 valves from 20 patients) is provided in Table 2 (and Supplementary Table S1, also including Gram staining).

**Table 2:** Yield of standard workup and sonication/centrifugation in positively tested heart valves from patients with active endocarditis (22 valves from 18 patients).

Pt nr	Heart valve	AB days	Blood culture prior to surgery	Standard		Sonication/centrifugation		Valve
				Culture (C) Plates (P)	16S-PCR	Culture (C) Plates (P)	16S-PCR	
1	N-AV	4	*Staphylococcus aureus*	C: 2+, P: all pos	pos	C: 2+, P: all pos	pos	*Staphylococcus aureus*
2	Bio P-PV	25	*Streptococcus parasanguinis*	neg	pos	neg	pos	*Streptococcus* spp.
2	Bio P-AV	25	*Streptococcus parasanguinis*	neg	pos	neg	pos	*Streptococcus* spp.
3	N-TV*	27	*Haemophilus parainfluenzae*	neg	neg	neg	pos	*Haemophilus parainfluenzae*
4	Bio P-AV*	19	neg	neg	pos	C: 58 CFU, P: BBA pos	neg	*Propionibacterium acnes*
11	Mech P-AV	0	neg	C: 1+, P: BBA pos	pos	C: 2+, P: BBA pos	pos	*Propionibacterium acnes*
14	N-PV	54	neg^†^	neg	pos	neg	neg	*Tropheryma whipplei*
14	N-AV	54	neg^†^	neg	pos	neg	neg	*Tropheryma whipplei*
29	Mech P-AV*	28	neg	neg	pos	C: 1 CFU, P: BBA pos	pos	*Propionibacterium acnes*
30	Bio P-AV*	0	neg	C: 1 CFU, P: BBA pos	neg	C: 2 CFU, P: BBA pos	neg	*Propionibacterium acnes*
				C: 2 CFU, P: BA pos	neg	C: 1 CFU, P: CHOC pos	neg	*Staphylococcus capitis*
32	N-AV	38	*Enterococcus faecalis*	neg	pos	neg	pos	*Enterococcus faecalis*
32	N-MV	38	*Enterococcus faecalis*	neg	pos	neg	neg	*Enterococcus faecalis*
46	Bio P-AV*	7	*Propionibacterium acnes*	neg	neg	C: 9 CFU, P: BBA pos	neg	*Propionibacterium acnes*
47	N-AV	27	*Staphylococcus dysgalactiae*	neg	pos	neg	pos	*Staphylococcus dysgalactiae*
48	N-AV	26	*Streptococcus mitis*	neg	pos	neg	pos	*Streptococcus mitis*
49	N-AV	25	*Streptococcus salivarius*	neg	pos	neg	neg	*Streptococcus salivarius*
51	Bio P-AV	0	neg	C: 20 CFU, P: BBA pos	neg	C: 76 CFU, P: BBA pos	neg	*Propionibacterium acnes*
52	N-AV*	15	*Enterococcus faecalis*	C: 1+, P: BBA	neg	C: 4 CFU, P: BA	pos	*Enterococcus faecalis*
			neg	C: 1+, P: BBA	neg	C: 6 CFU, P: BBA	neg	*Propionibacterium acnes*
52	Bio P-MV*	15	*Enterococcus faecalis*	C: 1+, P: BBA	neg	C: 2 CFU, P: BBA	neg	*Propionibacterium acnes*
55	N-AV	31	*Staphylococcus aureus*	neg	pos	neg	neg	*Staphylococcus aureus*
56	N-AV	29	*Streptococcus gordonii*	neg	pos	neg	pos	*Streptococcus gordonii*
58	Mech P-AV*	19	*Propionibacterium acnes*	neg	pos	C: 22 CFU, P: BBA	pos	*Propionibacterium acnes*

*Clinically useful diagnostic information added by sonication/centrifugation; ^†^*Tropheryma whipplei* identified with PCR on full blood; 1+: 10–100 CFU; 2+: 100–1000 CFU; 3+: >1000 CFU; AB days = number of days on antibiotic therapy prior to surgery; Bio = biological; CFU = colony forming unit; Mech = mechanical; N-AV = native aortic valve; neg = negative; N-MV = native mitralis valve; N-PV = native pulmonary valve; N-TV = native tricuspid valve; P-AV = prosthetic aortic valve; plates = solid media including BA, CHOC, and BBA plates; pos = positive; P-PV = prosthetic pulmonary valve; pt nr = patient number; Standard = standard workup, including direct culture on solid media (BA, CHOC, and BBA plates); Sonication/centrifugation: the sonication procedure of the explanted heart valve where after centrifugation of sonication fluid and thereafter culture of the sediment on solid media (BA, CHOC, and BBA plates).

**Figure 4:**
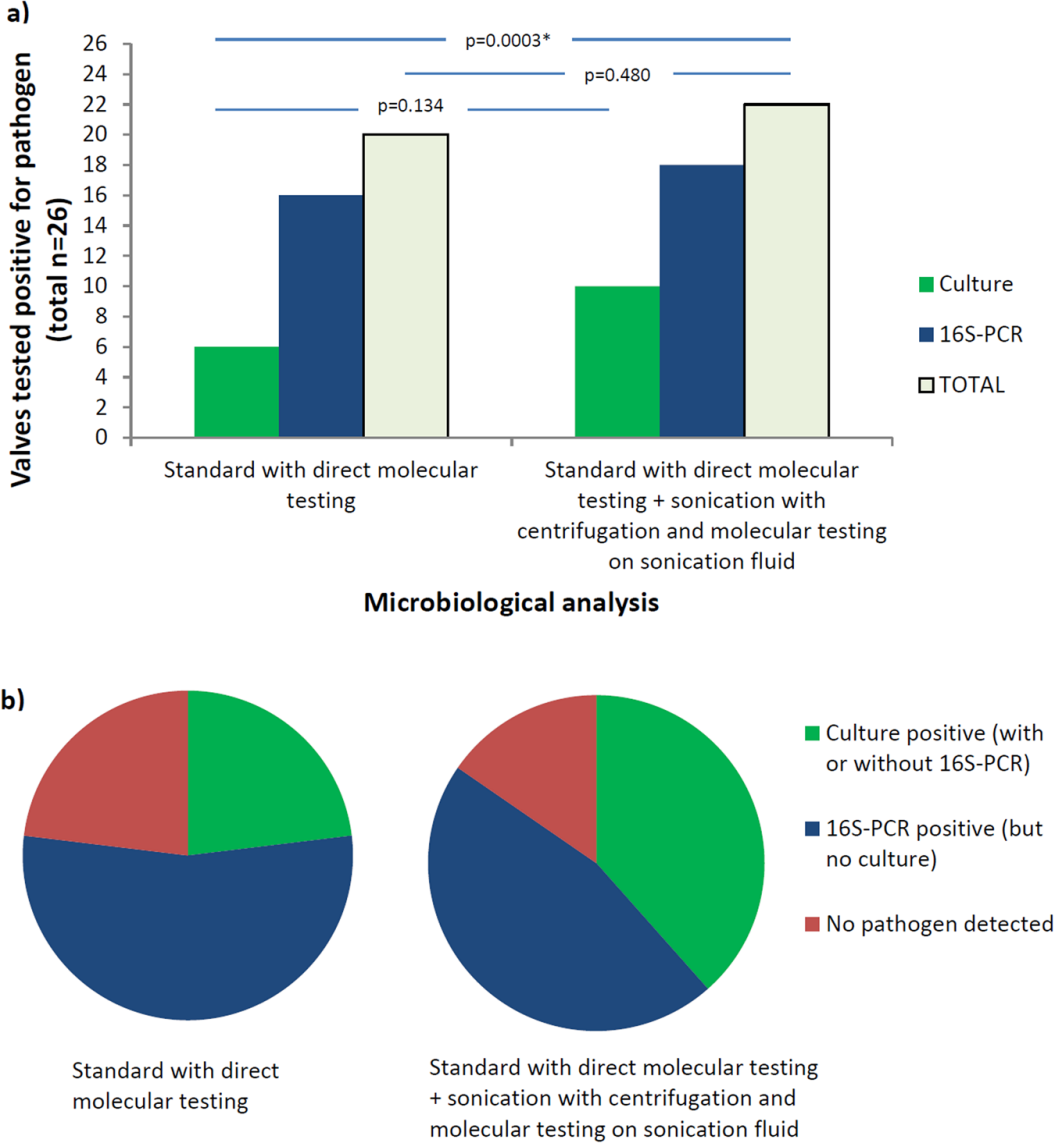
Heart valves (n = 26 in total) from patients with active endocarditis tested positive for culture and/or 16S-PCR. (**a**) Absolute number of valves – positive valves are counted separately for culture, 16S-PCR and in total, and thus one valve can be counted multiple times, (**b**) relative number of valves – n = 26 valves in each circle with each valve counted once for the qualitative best possible identification of its pathogen: 1. Culture, qualitatively the best option providing identification of the microorganism and antimicrobial susceptibility testing; 2. 16S-PCR, qualitatively the least option providing identification of the microorganism, only.

The total number of heart valves from patients with active definite endocarditis that tested positive for standard workup in total (n = 20) and in culture specifically (n = 6), versus those that tested positive for standard workup with direct molecular testing and sonication/centrifugation in total (n = 22) or in culture specifically (n = 10), is provided in Fig. 4a. Yield of culture (green bar) and 16S-PCR (blue bar) are specified separately. Sonication/centrifugation increased the total number of heart valves from patients with endocarditis in which the pathogen could be detected by culture and/or 16S-PCR (greyish green bar) from 20/26 to 22/26 (p = 0.480). Furthermore, it almost doubled the total number of these heart valves in which the pathogen could be cultured, allowing for antimicrobial resistance testing, from 6/26 to 10/26 (p = 0.134). Compared to direct culture only, the addition of sonication/centrifugation significantly increased the yield of microbiological testing from 6/26 to 17/26 (p = 0.003). Furthermore, addition of both sonication/centrifugation and 16S-PCR performed directly on the valve increased the yield of microbiological testing even more, from 6/26 to 22/26 (p = 0.0002). Figure 4b displays the relative proportion of heart valves in which the pathogen could be identified (blue plus green) and cultured (green only) with the addition of sonication/centrifugation to the standard workup.

### Prosthetic versus native valves

Combined standard workup with molecular testing and sonication/centrifugation, detected pathogens in prosthetic (10/11; 91%) and native (12/15;80%) heart valves. Sonication/centrifugation added extra information for microbiological diagnosis of infective endocarditis in 6 prosthetic heart valves (5 from patients who received prior antimicrobial therapy) and 2 native heart valve (2 from patients who received prior antimicrobial therapy).

### Potential source of *P. acnes* as contaminant

WGS was performed on the first 20 isolates of *P*. *acnes* from 11 patients. Most isolates were genetically highly diverse (Supplementary Fig. S1). Related strains were always isolated from the same patient. No correlation was found with the involved surgeons or laboratory technicians.

## Discussion

To the best of our knowledge, this is the first study focusing on the added value of sonication to standard workup of heart valves in infective endocarditis. Two different protocols of sonication were compared in this study. Sonication/enrichment yielded a surplus of false positive results in negative control valves (Fig. 3). Consequently, sonication/centrifugation of heart valves was selected as the method of choice. We showed that sonication/centrifugation added to direct culture significantly increased the diagnostic yield. Most importantly, culture positives almost doubled, suggesting that also without 16S-PCR sonication is of added value. This is in line with a previous study, showing increased culture yield by sonication (without centrifugation) investigating 8 infected heart valves^27^. In case of direct molecular testing in the standard workup, sonication/centrifugation still provided additional diagnostic information to the microbiological diagnosis of infective endocarditis in a substantial number of valves (Table 2 and Fig. 4). Added value was found in the diagnostic workup of both prosthetic and native valves, and both patients treated with and without antimicrobial therapy prior to surgery, suggesting that its highest value is in the diagnostic workup of patients with prosthetic valve endocarditis pretreated with antimicrobial therapy. Furthermore, sonication/centrifugation provided relevant information to determine the duration of therapy.

As many centers do not routinely include 16S-PCR on primary specimens in their standard workup, we analysed the added diagnostic yield of sonication/centrifugation to direct culture alone. This added diagnostic yield was particularly high (from 6/26 to 17/26, p = 0.003). Adding both sonication/centrifugation and 16S-PCR on primary specimens to direct culture alone increased the diagnostic yield of the microbiological workup even more (from 6/26 to 22/26, p = 0.0002). In this study, four valves tested positive only in 16S-PCR directly on the valve, and one valve tested positive only in 16S-PCR on sonication fluid. The addition of both sonication and molecular testing to direct culture results in a significantly higher diagnostic yield.

Potentially important information provided by sonication, besides identification of a pathogen in the first place, is culture of a pathogen. Culture is the ultimate diagnostic method as it provides unique quantitative information on the pathogen (species), information about its antimicrobial susceptibility pattern (allowing phenotypic resistance testing, including determination of the minimum inhibitory concentration [MIC] for pharmacokinetic/pharmacodynamics [PK/PD]-based therapy), and information about its viability. Moreover, culture after sonication might support a previously found microorganism as a true pathogen, and sonication might decrease time to positivity (allowing earlier implementation of adequate antimicrobial therapy). All of these data have most likely impact on patient management, including appropriate antimicrobial treatment.

Strengths of this study are its prospective design, the inclusion of a high number of negative control valves, the head-to-head comparison of two sonication protocols as well as 16S-PCR, and the NGS performed to analyse the possible origin of *P*. *acnes*. Important sources of bias were prevented, as a random sample of negative control valves and only consecutive cases with active definite endocarditis were included. In addition, results of sonication and standard workup were assessed independently of each other and sonication results were never part of the definite microbiological diagnosis of infective endocarditis. We chose to focus on the results of sonication/centrifugation in valves of active endocarditis patients based on a better performance of this method in the identification and culture of microorganisms, although sonication/enrichment was also performed in the first 17 valves (Supplementary Table S1).

A limitation of this study is its relatively low number of included infected valves, precluding subgroup analysis for native *vs*. prosthetic valves. A potential limitation of this study is that selected pathological/aberrant looking parts of the heart valve were used in the standard workup for culture on agar plates, but the same material would ideally have been used for 16S-PCR and sonication. Clearly, this ideal situation is impossible to achieve and we chose not to interfere with the standard workup. This conservative diagnostic workflow probably results in an underestimated diagnostic yield of sonication, suggesting that the added value of sonication/centrifugation could be intrinsically even higher. Limitations also include a lack of reliable knowledge about the antimicrobial therapy prescribed by the general practitioner, and the antimicrobial use as is mentioned in Table 2 concerns categorical information and did not take the activity against the found pathogen into account. Finally, although this study includes a large number of negative control valves, we do not have quantitative data on the methodological contamination error of sonication for prosthetic valves as only 2 were included in this investigation (Fig. 1).

Strikingly, we found a high portion of heart valves including negative controls, testing positive for *P*. *acnes*. In this study, total microbiological workup revealed this Gram-positive, facultative anaerobic rod in 16 valves: 9 from patients with endocarditis and 7 from patients serving as negative controls. For the 9 cases, there was no doubt about the diagnosis of infective endocarditis. For the 7 negative controls, *P*. *acnes* represented most likely contamination. This is consistent with the general interpretation, as this microorganism is a normal component of skin flora^28^. To elaborate this matter further, we performed NGS to investigate the genetic relatedness of the first isolated strains. The isolated *P*. *acnes* seemed to originate from the patients, excluding technical problems. This suggests that these organisms come from deeper skin layers which are accessible during cardiothoracic surgery.

Contamination is a risk for sonication, as shown in the investigation of negative control valves. As endocarditis is a complex infection, it requires careful interpretation and continuous evaluation of obtained results. For the centrifugation method it was possible to identify contamination based on quantification (number of CFU and number of positive agar plates and/or positive 16S-PCR). Therefore, we advise to be especially suspicious of contamination if <5 CFU of *P*. *acnes* (or potentially other low-grade pathogens) are cultured from sonication fluid and/or if no other media are positive (sonication, standard workup, previous blood cultures). This work-up is similar to that reported for the validated approach in prosthetic joint infections^18^.

In summary, this diagnostic proof of concept study demonstrates that sonication could be a valuable addition to the standard microbiological workup of heart valves. Future studies are needed to confirm that sonication/centrifugation of heart valves should be granted a solid place in the microbiological diagnostic workup of infective endocarditis and different sonication protocols should be evaluated. In this study, particularly in combination with molecular testing, sonication significantly increased the total number of valves in which a microorganism could be identified. An important further benefit was an increased number of positive cultures, providing relevant additional diagnostic information for optimal therapy.

## Electronic supplementary material

Supplementary Information - Figure S1, Tables S1 and S2.

## Data Availability

All data generated or analysed during this study are included in this published article (and its Supplementary Information files).

## References

[1] Saby L (2013). Positron emission tomography/computed tomography for diagnosis of prosthetic valve endocarditis: increased valvular ^18^F-fluorodeoxyglucose uptake as a novel major criterion. J. Am. Coll. Cardiol..

[2] Vos FJ, Bleeker-Rovers CP, Kullberg BJ, Adang EM, Oyen WJ (2011). Cost-effectiveness of routine ^18^F-FDG PET/CT in high-risk patients with gram-positive bacteremia. J. Nucl. Med..

[3] Thuny F, Grisoli D, Collart F, Habib G, Raoult D (2012). Management of infective endocarditis: challenges and perspectives. Lancet.

[4] Rohacek M (2015). Infection of cardiovascular implantable electronic devices: detection with sonication, swab cultures, and blood cultures. Pacing Clin. Electrophysiol..

[5] Inacio RC (2015). Microbial diagnosis of infection and colonization of cardiac implantable electronic devices by use of sonication. Int. J. Infect. Dis..

[6] Oliva A (2013). Sonication of explanted cardiac implants improves microbial detection in cardiac device infections. J. Clin. Microbiol..

[7] Cahill TJ (2017). Challenges in Infective Endocarditis. J Am Coll Cardiol.

[8] Vinh DC, Embil JM (2005). Device-related infections: a review. J. Long. Term. Eff. Med. Implants.

[9] Taraszkiewicz, A., Fila, G., Grinholc, M. & Nakonieczna, J. Innovative strategies to overcome biofilm resistance. *Biomed*. *Res*. *Int*., 10.1155/2013/150653. (2013). 10.1155/2013/150653 PMC3591221 23509680

[10] Mah TF, O’Toole GA (2001). Mechanisms of biofilm resistance to antimicrobial agents. Trends Microbiol..

[11] Mason PK (2011). Sonication of explanted cardiac rhythm management devices for the diagnosis of pocket infections and asymptomatic bacterial colonization. Pacing Clin. Electrophysiol..

[12] Trampuz A (2007). Sonication of removed hip and knee prostheses for diagnosis of infection. N. Engl. J. Med..

[13] Pitt WG, Ross SA (2003). Ultrasound increases the rate of bacterial cell growth. Biotechnol. Prog..

[14] Holinka J (2011). Sonication cultures of explanted components as an add-on test to routinely conducted microbiological diagnostics improve pathogen detection. J. Orthop. Res..

[15] Piper KE (2009). Microbiologic diagnosis of prosthetic shoulder infection by use of implant sonication. J. Clin. Microbiol..

[16] Tunney MM (1998). Improved detection of infection in hip replacements. A currently underestimated problem. J. Bone Joint Surg. Br..

[17] Yano MH (2014). Improved diagnosis of infection associated with osteosynthesis by use of sonication of fracture fixation implants. J. Clin. Microbiol..

[18] Zhai Z (2014). Meta-analysis of sonication fluid samples from prosthetic components for diagnosis of infection after total joint arthroplasty. J. Clin. Microbiol..

[19] Oliva A (2016). Role of Sonication in the Microbiological Diagnosis of Implant-Associated Infections: Beyond the Orthopedic Prosthesis. Adv. Exp. Med. Biol..

[20] Oliva A (2010). Pacemaker lead endocarditis due to multidrug-resistant Corynebacterium striatum detected with sonication of the device. J. Clin. Microbiol..

[21] Rohacek M (2010). Bacterial colonization and infection of electrophysiological cardiac devices detected with sonication and swab culture. Circulation.

[22] Nagpal A (2015). Usefulness of sonication of cardiovascular implantable electronic devices to enhance microbial detection. Am. J. Cardiol..

[23] Li JS (2000). Proposed modifications to the Duke criteria for the diagnosis of infective endocarditis. Clin. Infect. Dis..

[24] Benson DA (2002). GenBank. Nucleic Acids Res..

[25] Ferdous M, Kooistra-Smid AM, Zhou K, Rossen JW, Friedrich AW (2016). Virulence, Antimicrobial Resistance Properties and Phylogenetic Background of Non-H7 Enteropathogenic Escherichia coli O157. Front. Microbiol..

[26] Zhou K (2015). The mosaic genome structure and phylogeny of Shiga toxin-producing Escherichia coli O104:H4 is driven by short-term adaptation. Clin. Microbiol. Infect..

[27] Oberbach, A. *et al*. New insights into valve-related intramural and intracellular bacterial diversity in infective endocarditis. *Plos One* **12**, 10.1371/journal.pone.0175569 (2017). 10.1371/journal.pone.0175569 PMC5391965 28410379

[28] Achermann Y, Goldstein EJ, Coenye T, Shirtliff ME (2014). Propionibacterium acnes: from commensal to opportunistic biofilm-associated implant pathogen. Clin. Microbiol. Rev..

