# Supplementary material for: Sonication of heart valves detects more bacteria in infective endocarditis
Source: Sci Rep. 2018 Aug 28;8:12967. doi: 10.1038/s41598-018-31029-w (PMC6113321; doi:10.1038/s41598-018-31029-w)
Supplement: Supplementary file 1 — Supplementary Information - Figure S1, Tables S1 and S2. [file 41598_2018_31029_MOESM1_ESM.pdf]

Sonication of heart valves

detects more bacteria in infective endocarditis

Anna Gomes*, MD[1]; Marleen van Oosten, MD PhD[1]; Kasper L.B. Bijker, MD[1]; Kathleen E. Boiten[1]; Elisa N. Salomon[1]; Sigrid Rosema[1]; John W.A. Rossen, PhD[1]; Ehsan Natour, MD PhD[2]; Yvonne L. Douglas, MD PhD[3]; Greetje A. Kampinga, MD PhD[1]; Sander van Assen, MD PhD[4]; Bhanu Sinha, MD PhD[1]

[1]Department of Medical Microbiology and Infection Prevention, University of Groningen, University Medical Center Groningen, Groningen, the Netherlands; [2]Department of Thoracic Surgery, Maastricht University Medical Center, Maastricht, the Netherlands; [3]Department of Cardio-Thoracic Surgery, University of Groningen, University Medical Center Groningen, Groningen, the Netherlands; [4]Department of Internal Medicine (Infectious Diseases), Treant Zorggroep, Hoogeveen, the Netherlands.

*Correspondence: Anna Gomes, University Medical Center Groningen, Department of Medical Microbiology, HPC: EB 80, Hanzeplein 1, 9713 GZ Groningen, the Netherlands. E-mail: a.gomes@umcg.nl; Telephone: +31 50 3617533; Fax + 31 50 3619105.

Supplementary Figure S1: **Ridom SeqSphere+ minimum spanning trees based on 1874 core genome multilocus sequence typing targets with pairwise ignoring missing values and a logarithmic scale.** Colours in circles = individual patients, red dashed line around circle = isolate retrieved from patient with active endocarditis, white circle = reference strain HL096PA1 used to define the core genome multilocus sequence typing scheme**,** S1-19 = strains/isolates, 1-5 = surgeon, A-F = laboratory technician. Numbers between the circles indicate the number of different alleles (one or more single nucleotide polymorphisms) between strains. Note: the graphic distance between dots does NOT indicate their genetic distance; the latter are indicated by the numbers (of different single nucleotide polymorphisms).

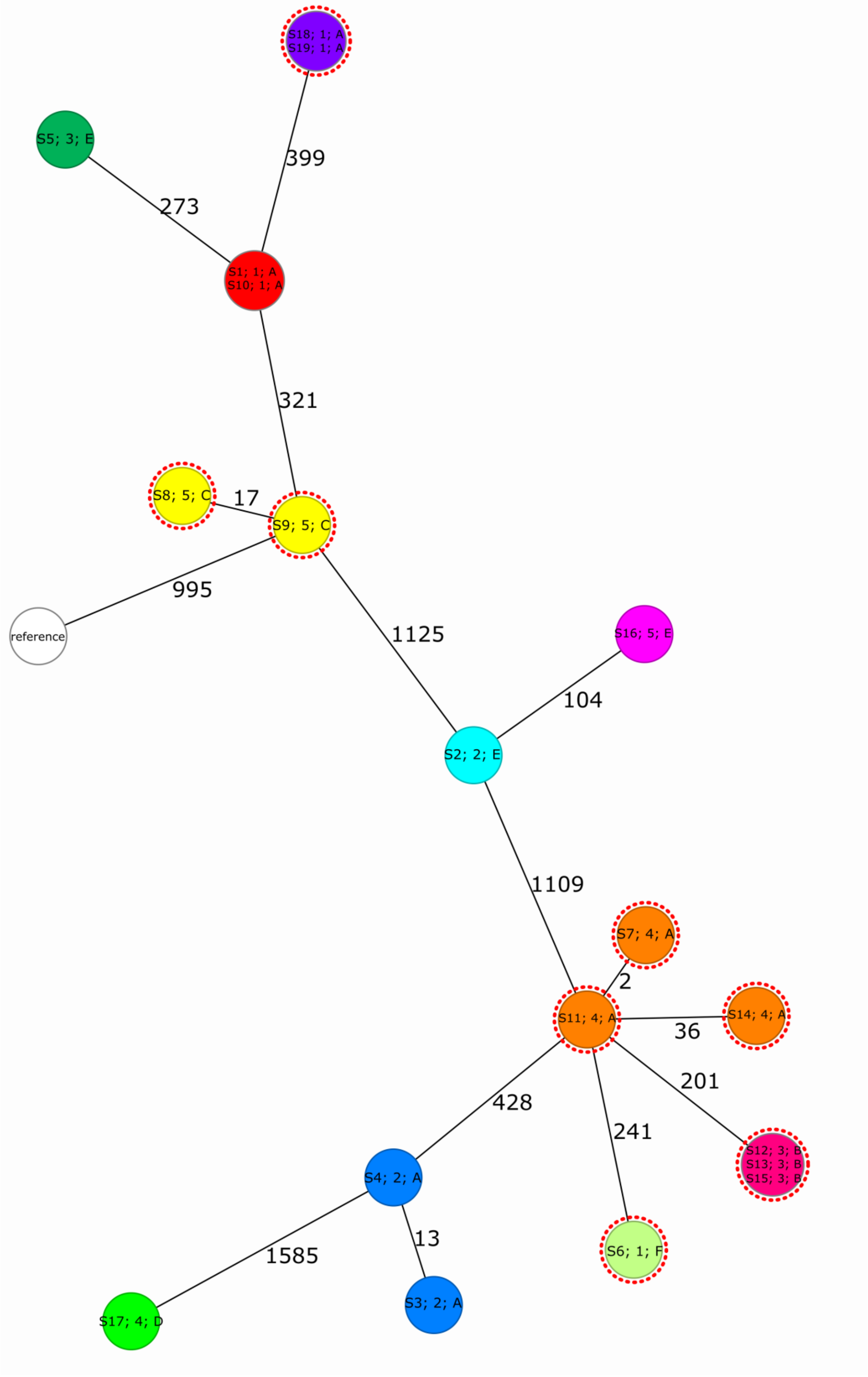

***Supplementary Table S1:* Yield of positive heart valves (22 valves from 18 patients with endocarditis and 11 valves from 11 patients without endocarditis) in standard and sonication methods.** *Clinically useful diagnostic information added by the sonication centrifugation method. *(*)* = *Tropheryma whipplei* identified with PCR on full blood | 1+: 10-100 CFU | 2+: 100-1000 CFU | 3+: >1000 CFU | AB days = number of days on antibiotic therapy prior to surgery | BA = sheep-blood agar | BBA = *Brucella* blood agar | BC = blood culture(s) | Bio = biological | C = centrifugation, including centrifugation of sonication fluid and thereafter culture of the sediment on solid media (BA, CHOC, and BBA plates) | CFU = colony forming unit(s) | CHOC = Chocolate agar | E = enrichment, including direct culture of sonication fluid on solid media (BA, CHOC, and BBA plates) as well as enrichment of sonication fluid in blood culture bottles | *E. faecalis* = *Enterococcus faecalis* | Gram = Gram staining | *H. parainfluenzae* = *Haemophilus parainfluenzae* | M(D) = molecular testing, in this study compromising 16S-PCR performed directly on aberrant looking parts of the valve | Mech = mechanical | M(S) = molecular testing, in this study compromising 16S-PCR performed on the sediment of sonication fluid retrieved after centrifugation | N-AV = native aortic valve | N-MV = native mitralis valve | N-PV = native pulmonary valve | N-TV = native tricuspid valve | *P. acnes* = *Propionibacterium acnes* | P-AV = prosthetic aortic valve | P-PV = prosthetic pulmonary valve | pt nr = patient number | S = sonication, includes the sonication procedure of the explanted heart valve, hereafter the sonication fluid was handled according to two different protocols (enrichment and centrifugation) | *S. aureus* = *Staphylococcus aureus* | *S. capitis* = *Staphylococcus capitis* | *S. dysgalactiae* = *Streptococcus dysgalactiae* | *S. epidermidis* = *Staphylococcus epidermidis* | *S. gordonii* = *Streptococcus gordonii* | *S. mitis* = *Streptococcus mitis* | *S. parasanguinis* = *Streptococcus parasanguinis* | *S. pneumoniae* = *Streptococcus pneumoniae* | *S. saccharolyticus* = *Staphylococcus saccharolyticus* | *S. salivarius* = *Streptococcus salivarius* | ST = standard work-up, including Gram and direct culture on solid media (BA, CHOC, and BBA agar plates) | TTP = time to positivity | *T. whipplei* = *Tropheryma whipplei* | 16S-PCR = 16S ribosomal DNA gene amplification analysis.

Pt nr	Heart valve	AB days	Blood culture prior to surgery	ST		S/E		S/C		Valve
				Culture/ GRAM	M(D): 16S-PCR	Culture	E: BC (TTP)	Culture/ GRAM	M(S): 16S-PCR	
Active endocarditis										
1	N-AV	4	*S. aureus*	BA/ CHOC/ BBA 2+, Gram	+	BA/ CHOC/ BBA 2+	>9.9hr	BA/ CHOC/ BBA 2+, Gram	+	*S. aureus*
2	Bio P-PV	25	*S. parasanguinis*	-	+	-	-	-	+	*Streptococcus* spp.

2	Bio P-AV	25	*S. parasanguinis*	-	+	-	-	-	+	*Streptococcus* spp.
3	**N-TV***	27	*H. parainfluenzae*	-	-	-	-	-	+	*H. parainfluenzae*
4	**Bio P-AV***	19	**-**	Gram	+	BBA 4 CFU	>255.9 hr	BBA 1+	-	*P. acnes*
11	Mech P-AV	0	-	BBA 1+	+	BBA 2+	>83.2 hr	BBA 2+	+	*P. acnes*
14	N-PV	54	- *(*)*	-	+	-	-	-	-	*T. whipplei*
14	N-AV	54	- *(*)*	-	+	-	-	-	-	*T. whipplei*
29	**Mech P-AV***	28	**-**	-	+	-	-	BBA 1 CFU	+	*P. acnes*
30	**Bio P-AV***	0	**-**	BBA 1 CFU	-	-	-	BBA 2 CFU	-	*P. acnes*
				BA 2 CFU	-	-	-	CHOC 1 CFU	-	*S. capitis*
32	N-AV	38	*E. faecalis*	-	+	-	-	-	+	*E. faecalis*
32	N-MV	38	*E. faecalis*	-	+	-	-	-	-	*E. faecalis*
46	**Bio P-AV***	7	*P. acnes*	Gram	-	BBA 1 CFU	-	Gram, BBA 9 CFU	-	*P. acnes*
47	N-AV	27	*S. dysgalactiae*	Gram	+	-	-	Gram	+	*S. dysgalactiae*
48	N-AV	26	*S. mitis*	Gram	+			Gram	+	*S. mitis*
49	N-AV	25	*S. salivarius*	Gram	+			-	-	*S. salivarius*
51	Bio P-AV	0	-	BBA 20 CFU	-			BBA 76 CFU	-	*P. acnes*
52	**N-AV***	15	*E. faecalis*	BBA 1+	-			Gram, BA 4 CFU	+	*E. faecalis*
			-	BBA 1+	-			BBA 6 CFU	-	*P. acnes*
52	**Bio P-MV***	15	*E. faecalis*	BBA 1+	-			BBA 2 CFU	-	*P. acnes*
55	N-AV	31	*S. aureus*	-	+			-	-	*S. aureus*
56	N-AV	29	*S. gordonii*	-	+			-	+	*S. gordonii*

58	**Mech P-AV***	19	*P. acnes*	-	+			Gram, BBA 22 CFU	+	*P. acnes*
Controls										
13	N-AV	0	-	-	-	-	179 hr	-	-	*P. acnes*
18	Bio P-PV	0	-	-	-	-	32.8 hr	-	-	*S. epidermidis*
20	N-AV	0	-	-	-	-	139.8 hr	-	-	*P. acnes*
21	N-AV	0	-	-	-	-	-	BBA 1 CFU	-	*P. acnes*
23	N-AV	0	-	-	-	-	101.5 hr	-	-	*P. acnes*
26	N-AV	0	-	-	-	BBA 2 CFU	>178.9 hr	-	-	*P. acnes*
28	N-AV	0	-	-	-	BBA 2+	-	-	-	*P. acnes*
34	N-AV	0	-	-	-	-	109.8 hr	BBA 1 CFU	-	*P. acnes*
36	N-AV	0	-	BBA 1CFU	-	BBA 1 CFU	-	-	-	*S. pneumoniae*
				-	-	-	-	BBA 1 CFU	-	*S. epidermidis*
40	N-AV	0	-	-	-	-	22.7 hr	-	-	*S. epidermidis*
45	N-AV	0	-	BBA 3 CFU	-	-	92.7 hr	BBA 6 CFU	-	*S. saccharolyticus*

Supplementary Table S2: **Pathological examination of negative control valves.** Grey coloured lines display information about valves tested positive for any microorganism. Abbreviations: AoS = aortic valve stenosis, CAD = coronary artery disease, LCC = left coronary cusp, NCC = non coronary cusp, PAF = paroxysmal atrial fibrillation, RCC = right coronary cusp, VSD = ventricular septum defect.

**Negative control study number**		**Pathological examination**
**Valve**	**Patient**	
0023	0005	Diagnosis/indication for surgery: AoS and aorta ascendens aneurysm. Quality aorta: moderate. Aortic valve morphology: 2 cusps. Aortic valve state: calcified.
0025	0006	Diagnosis/indication for surgery: severe AoS. Quality aorta: moderate, severely calcified aortic root and wall. Aortic valve morphology: 3 cusps. Aortic valve state: calcified.
0026	0007	Diagnosis/indication for surgery: AoS.<br>Quality aorta: reasonable. Aortic valve morphology: 3 cusps, fusion of RCC and LCC. Aortic valve state: calcified.
0028	0008	Diagnosis/indication for surgery: CAD and severe AoS. Aortic valve morphology: 2 cusps, fusion of LCC and NCC. Aortic valve state: severely sick and calcified, with calcification extending towards mitral valve cusp.
0029	0009	Diagnosis/indication for surgery: AoS. Quality aorta: reasonable. Aortic valve morphology: 3 cusps.<br>Aortic valve state: calcified.
0030	0010	Diagnosis/indication for surgery: severe AoS and severe CAD. Quality aorta: good. Aortic valve morphology: 3 cusps. Aortic valve state: calcified.
0040	0013	Diagnosis/indication for surgery: AoS. Quality aorta: reasonable. Aortic valve morphology: 3 cusps. Aortic valve state: calcified, calcified fusion LCC and RCC.
0044	0015	Diagnosis/indication for surgery: severe AoS. Quality aorta: poor quality of aorta ascendens resulting from severe calcifications. Aortic valve morphology: 3 cusps. Aortic valve state: calcified.
0045	0016	Diagnosis/indication for surgery: AoS, CAD, PAF, mitral valve insufficiency. Quality aorta: reasonable. Aortic valve morphology: 3 cusps. Aortic valve state: calcified.
0047	0017	Diagnosis/indication for surgery: AoS. Quality aorta: good. Aortic valve morphology: 3 cusps. Aortic valve state: calcified.
0048	0018	Diagnosis/indication for surgery: state after correction pulmonary valve atresia and VSD, multiple reoperation for pulmonary valve replacements, at present pulmonary valve stenosis and insufficiency of implanted Contegra conduit with right ventricle dilatation for which fifth reoperation replacing the pulmonary valve with a homograft.
0049	0019	Diagnosis/indication for surgery: AoS. Quality aorta: reasonable. Aortic valve morphology: 3 cusps. Aortic valve state: calcified.
0050	0020	Diagnosis/indication for surgery: AoS. Quality aorta: moderate. Aortic valve morphology: 3 cusps. Aortic valve state: calcified.
0051	0021	Diagnosis/indication for surgery: severe AoS and de novo PAF. Quality aorta: reasonable. Aortic valve morphology: 2 cusps, bicuspid valve with

		a raphe between LCC and RCC. Aortic valve state: severely calcified, extending towards anterior leaflet of mitral valve.
0052	0022	Diagnosis/indication for surgery: AoS. Quality aorta: poor. Aortic valve morphology: 3 cusps. Aortic valve state: dilated, calcified. Additional note: prosthetic aortic valve sized for 23 mm, but unable to position properly below left coronary artery ostium because of tearing and very fragile aorta. Therefore a 21 mm valve was placed without annuloplasty because of the very fragile tissue.
0054	0023	Diagnosis/indication for surgery: AoS. Quality aorta: good. Aortic valve morphology: 3 cusps. Aortic valve state: calcified.
0055	0024	Diagnosis/indication for surgery: severe AoS. Aortic valve morphology: 3 cusps. Aortic valve state: calcified.
0056	0025	Diagnosis/indication for surgery: AoS, CAD, mitral valve insufficiency, interstitial pulmonary disease *e causa ignota*. Quality aorta: reasonable. Aortic valve morphology: 3 cusps. Aortic valve state: calcified.
0057	0026	Diagnosis/indication for surgery: AoS, CAD. Quality aorta: moderate. Aortic valve morphology: 3 cusps. Aortic valve state: calcified.
0058	0027	Diagnosis/indication for surgery: severe AoS, CAD. Quality aorta: reasonable. Aortic valve morphology: 3 cusps. Aortic valve state: calcified.
0063	0028	Diagnosis/indication for surgery: state after aortic valve plasty and valve saving root replacement, with afterwards recurrent aortic valve insufficiency. Aortic valve inspection: calcified, the plication sutures are torn.
0072	0031	Diagnosis/indication for surgery: symptomatic AoS. Aortic valve morphology: 3 cusps. Aortic valve state: calcified.
0075	0033	Diagnosis/indication for surgery: patient-prosthesis mismatch with a state after aortic valve replacement and dilation plasty according to Manouguian using autologous pericardium. Aortic valve inspection: extensive pannus overgrowing the inflow side of the prosthetic valve and obstructing normal movement of the leaflets.
0076	0034	Diagnosis/indication for surgery: AoS. Quality aorta: moderate. Aortic valve morphology: 3 cusps. Aortic valve state: calcified.
0077	0035	Diagnosis/indication for surgery: AoS and CAD in a patient on dialysis and with hypertension. Quality aorta: moderate. Aortic valve morphology: 3 cusps. Aortic valve state: calcified.
0078	0036	Diagnosis/indication for surgery: severe AoS and PAF. Quality aorta: reasonable. Aortic valve morphology: 3 cusps. Aortic valve state: calcified.
0079	0037	Diagnosis/indication for surgery: AoS, CAD. Quality aorta: moderate. Aortic valve morphology: 3 cusps. Aortic valve state: calcified.
0081	0038	Diagnosis/indication for surgery: severe AoS with bicuspid aortic valve and state after aortic coarctation resection via lateral sternotomy. Quality aorta: reasonable. Aortic valve morphology: 2 cusps. Aortic valve state: severely stenotic and calcified.
0082	0039	Diagnosis/indication for surgery: AoS. Quality aorta: reasonable. Aortic valve morphology: 3 cusps. Aortic valve state: calcified.
0083	0040	Diagnosis/indication for surgery: severe AoS with state after aortic coarctation resection and closing of VSD and right ventricular outflow tract obstruction repair. Aortic valve morphology: 2 cusps, bicuspid

		valve. Aortic valve state: severely stenotic with diffuse calcification.
0084	0041	Diagnosis/indication for surgery: AoS. Quality aorta: good. Aortic valve morphology: 3 cusps. Aortic valve state: calcified.
0085	0042	Diagnosis/indication for surgery: AoS, CAD. Quality aorta: poor. Aortic valve morphology: 3 cusps. Aortic valve state: calcified.
0088	0043	Diagnosis/indication for surgery: aortic valve stenosis/insufficiency. Quality aorta: reasonable. Aortic valve morphology: 3 cusps. Aortic valve state: calcified.
0089	0044	Diagnosis/indication for surgery: mitral valve stenosis. Morphology: valve and annulus calcified, with severe calcification of the posterior annulus. All chordae had to be sacrificed during surgery, replaced by three new chordae.
0090	0045	Diagnosis/indication for surgery: aortic valve stenosis/insufficiency. Quality aorta: reasonable. Aortic valve morphology: 2 cusps, in construction the valve was bicuspid with a raphe between LCC and RCC. Aortic valve state: calcified.
